# Clandestine abortions: epidemiology at Provincial General Hospital of Kananga

**DOI:** 10.11604/pamj.2022.42.320.34972

**Published:** 2022-08-29

**Authors:** Antoine Tshimbundu Kayembe, Gérard Kabatantshi Mukengabantu, Sylvain Mulumba Kapuku

**Affiliations:** 1 Department of Gynaecology and Obstetrics, Faculty of Medicine, University Notre-Dame of Kasayi, Central Kasaï, Democratic Republic of the Congo

**Keywords:** Epidemiology, clandestine abortions, Provincial General Hospital, Kananga

## Abstract

This study aim is to describe the epidemiological profile of pregnant women who performed clandestine abortions in the gynecological department of the Provincial General Hospital of Kananga. This is a descriptive study of a series conducted from the medical files of pregnant women who secretly aborted in the gynecology department of the Provincial General Hospital of Kananga from January 1^st^ 2015 to December 31^st^ 2019. It is based on the no probabilistic sampling of suitability. We recorded 58 cases of clandestine abortions upon 1667 patients. The frequency of clandestine abortions is of 3.48% with an average age of pregnant women of 26.96 years, 70% of pregnant women under 30 years old, nulliparity is more concerned (34.48%), pupils and students as well as unemployed are more concerned in 55.12%, nurses are the abortionists encountered in 37.70%, cervical dilation and curettage is the most used abortion method (69.07%) and the maternal mortality rate linked to clandestine abortion is 13.79%. The fight against clandestine abortions requires the promotion of sex education in school and academic circles, and the sensitization of women and couples to the use of contraceptive methods and family planning. Our results serve as the basis for in-depth studies on clandestine abortions and their complications with a view to improving their management in our environment.

## Introduction

Clandestine abortion is a voluntary termination of pregnancy under conditions not authorized by law. It has become rare in Western developed countries due to the legalization of induced abortion [1, 2]. It remains a public health problem in developing countries where legislation prohibits this practice [3, 4]. It is a source of morbidity and especially of maternal mortality in the world [1, 2].

According to the WHO, nearly 44 million induced abortions were performed worldwide in 2008, the rate is higher in developing countries than in industrialized countries (29 abortions per 1000 women aged 15-44 against 24 per 1000 respectively) [5]. Despite gynecological progress in recent years, clandestine abortion remains a silent drama that kills women every year [5]. Clandestine abortion is three times more deadly than therapeutic induced abortion [5]. Women desperate to end unwanted pregnancies often turn to clandestine “abortionists” in street clinics. Many of them use rudimentary and extremely dangerous techniques [5]. According to the WHO, 4.2 million unsafe abortions occur every year in Africa, resulting in nearly 300.000 deaths [5]. Globally, 44% of women who die from complications due to unsafe abortion are Africans [5].

The absence of local and recent statistics of clandestine abortions led us to conduct this study at the gynecology ward of the Provincial General Hospital (PGH) of Kananga, the aim of which is to describe the epidemiological profile of clandestine abortions at the PGH from January 1^st^ 2015 to December 31^st^ 2019.

## Methods

**Study design and setting:** this is a descriptive study of a series of cases conducted on the files of patients who had clandestinely aborted in the gynecology department of the PGH of Kananga from January 1^st^ 2015 to December 31^st^ 2019. The PGH was chosen at because of its situation as the 2^nd^ provincial reference hospital for cases, the presence of experienced staff and the high attendance of patients who have confidence in its staff.

**Study population:** we used the medical records of pregnant women aged between 15 and 44 years old, who had secretly aborted in the gynecology department of the PGH of Kananga, from January 1^st^ 2015 to December 31^st^ 2019.

**Sampling:** our sampling was non-probabilistic of suitability. The sample size was determined by the limitation of our study in time and space. The following criteria allowed us to include the pregnant women in the study: pregnant women aged between 16 and 45 years old having had a clandestine abortion in the gynecology department of the PGH of Kananga from January 1^st^ 2015 to December 31^st^ 2019 and whose medical file was complete. Incomplete medical records were excluded.

**Collection of data:** the data was collected from registers of the operating room, those of the gynecology department, medical records of patients from the gynecology department of the PGH of Kananga and the data collection record. The variables of the study are: year of study, age of pregnant women, parity, socio-professional status, marital status, qualification of abortionists, abortion methods and post-abortion follow-up.

**Statistical analyzes:** data were analyzed using Statistical Package for Social Sciences (SPSS) software version 20. We used the average (SD) to present the quantitative variables and the proportion to present the qualitative variables.

**Ethical considerations:** principles of medical ethics and documentary studies rules have been respected; the data were collected confidentially and treated anonymously.

## Results

**Figure 1:**
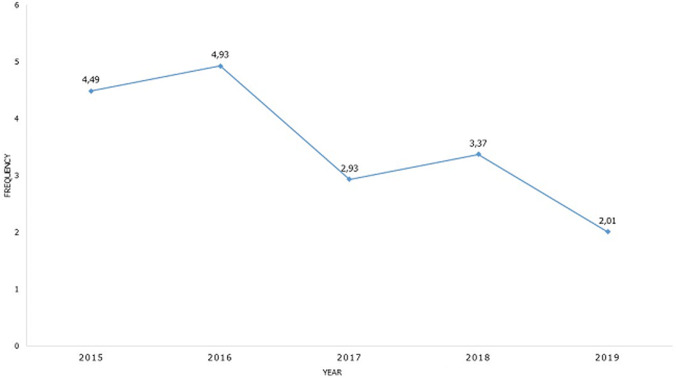
frequency’s evolution of clandestine abortion during our study’s period

**Frequency of clandestine abortions:** we recorded 58 clandestine abortions out of 1667 pregnant women in the gynecology department of the PGH of Kananga, i.e. a clandestine abortion frequency of 3.48%, the evolution of which during the period of our study is up and down starting from 4.49% in 2015 to 2.01% in 2019 passing by 4.93% in 2016. The annual average of clandestine abortions is 11.60 (SD 2.88) cases per year (Figure 1).

**Age and parity:** the age group most affected by clandestine abortions ranges from 25 to 29 years with 20 cases or 34.48%, 70% of pregnant women are under 30 years old and the average age of pregnant women with clandestine abortions is 25.96 (SD 6.33) years. Nulliparity is concerned in 20 cases or 34.48%, primiparity in 12 cases or 20.68%, pauciparity in 10 cases or 17.24%, multiparity in 10 cases or 17.24% and grand multiparity in 6 cases or 10.34%. The average parity is 4.05 (SD 2.50) (Table 1).

**Table 1:** distribution of cases according to age group and parity

Age range	N=58	%
15-19	10	17.24
20-24	14	24.14
25-29	20	34.48
30-34	7	12.07
35-39	6	10.35
40-44	1	1.73
**Parity**		
Nulliparity	20	34.48
Primiparity	12	20.68
Pauciparity	10	17.24
Multiparity	10	17.24
Grand multiparity	6	10.34

**Socio-professional status and marital status:** the unemployed had clandestine abortions in 10 cases, i.e. 17.24%, pupils and students in 22 cases or 37.93%, shopkeepers in 8 cases or 13.79%, civil servants in 8 cases or 13.79%, teachers in 6 cases or 10.34% and policewomen in 4 cases or 6.89%. As for marital status, singles are found in 22 cases or 37.97%, married in 10 cases or 17.24%, divorcees in 8 cases or 13.79%, widows in 6 cases or 10.34% and unspecified status in 12 cases or 20.68% (Table 2).

**Table 2:** distribution of cases according to socio-professional status and marital status

Statut professionnel	N=58	%
Unemployed	10	17.24
Pupils and students	22	37.93
Shopkeepers	8	13.79
Female teachers	6	10.34
Civil servant	8	13.79
Policewomen	4	6.89
**Marital status**		
Singles	22	37.97
Brides	10	17.24
Divorced	8	13.79
Widows	6	10.34
Not known	12	20.68

**Quality of abortionists, abortion methods and post-abortion follow-up:** doctors clandestinely aborted in 6 cases or 10.34%, nurses in 22 cases or 37.70%, traditional practitioners in 8 cases or 13.79%, the pregnant woman herself (self-abortion) in 4 cases or 6.89%, unidentified or unacknowledged abortionists in 18 cases or 31.03%. Traditional decoctions were used in 18 cases or 31.03% and cervical dilation - curettage in 40 cases or 69.07%. As for the post-abortion suites, they are simple with less than 10 days of hospitalization in 20 cases or 34.48%, complicated with 10 days and more of hospitalization in 30 cases or 51.72% and characterized by maternal death in 7 cases or 13.79% clandestine abortion (Table 3).

**Table 3:** distribution of cases according to qualification of abortionists, method of abortion and post-abortion follow-up

Qualification of abortionists	N	%
Doctors	6	10.34
Nurses	22	37.90
Tradittional practitioners	8	13.79
Pregnant herself (self-abortion)	4	6.89
Not identified or unacknowledged	18	31.03
**Abortion methods**		
Traditional decoctions	18	31.03
Cervical dilatation and curettage	40	69.07
Post-abortion suites		
Simple with less than 10 days of hospitalization	20	34.48
complicated with 10 days or more of hospitalization	30	51.72
Maternal death	8	13.79

## Discussion

We recorded 58 clandestine abortions out of 1667 pregnant women at the gynecology department of the PGH of Kananga, i.e. a frequency of clandestine abortions of 3.48%, the evolution of which during the period of our study is up and down starting from 4.49% in 2015 to 2.01% in 2019 through 4.93% in 2016. Our frequency figure is well below the 4.80% of Iloki *et al*. in Brazzaville [2] and the 6.50% of Megafu *et al*.in Nigeria in 1991 [6]. These high frequencies (≥1%) of clandestine abortions in our African communities are certainly linked to the extent of unwanted pregnancies secondary to the non-use or non-mastery of contraceptive methods [3]. Hence it is desirable to promote contraceptive methods in our communities. According to Hubinont in Brussels, the rate of induced abortions was higher than 1.00% in countries where these abortions are not legalized while it was 4 per 100.000 in those where they are legalized [7], a finding approved by the WHO [5]. This may also explain our frequency (greater than 1).

The age group most affected by clandestine abortions ranges from 25 to 29 years with 20 cases or 34.48% with an average age at clandestine abortions of 25.96 (SD 6.33) years. In the series by Mayi-Tsonga *et al*. in Libreville, the average age was 22.50 (SD 5.30) years [3, 10], which is slightly lower than ours. In many African studies [3, 4, 8-12], the age group most exposed to AC is between 19 and 25 years old. This is not the case in our case series. But abortion concerned all age groups between 16 and 45, i.e. the most sexually active women in our study environment. This observation was made by Mayi-Tsonga *et al*. in Libreville in 2008 [3]. About 70.00% of our pregnant women are under 30 years old. This corroborates the finding of Iloki *et al*. in Brazzaville which found that clandestine abortions preferentially concerned adolescent girls [2].

With regard to socio-professional status, pupils and students are the most exposed to clandestine abortion in 22 cases or 37.93% in our environment, followed by the unemployed in 10 cases or 17.24%. Our results meet those of Iloki *et al*. in Brazzaville where pupils and students were the most exposed to induced abortions [2]. Hence it is desirable to promote the school and academic education of girls in responsible sexuality in our environment as recommended by many other African authors [13-16].

As for marital status, single people are more affected by clandestine abortions in 22 cases or 37.97%. Our results are in agreement with those of the literature where Iloki *et al*. in Brazzaville reported more than 60.00% of unmarried pregnant women having clandestinely aborted [2]. Nulliparity is more concerned in 20 cases, i.e. 34.48% with an average parity of 4.05 (SD 2.50). This meets the results of the literature where Iloki *et al*. in Brazzaville reported the predominance of nulliparous in 49.00% [2].

As for the qualification of abortionists, nurses are the abortionists encountered in 22 cases, or 37.90% in our study environment. These results do not corroborate those of the literature where the pregnant women themselves were responsible (auto-abortionists) the most encountered for their own clandestine abortion in 69.80% of cases in Libreville [3] and 49.80% in Brazzaville [2]. The self-medication of pregnant women favored by the illicit sale of medicinal substances called “street drugs” which constitutes the most frequent cause in Brazzaville [2] and Libreville [3] was found in 6.89% of cases in our milieu.

With regard to the abortive methods, the traditional decoctions of nature ignored by the pregnant woman were used in 18 cases or 31.03% and cervical dilation-curettage in 40 cases or 69.07%. This means that cervical dilation and curettage is the most used abortion method in our study environment. In the case series of Mayi-Tsonga *et al*. in Libreville, it is the use of misoprostol which is the most used abortive method [3] whereas in that of Iloki *et al*. in Brazzaville, traditional decoctions are the most widely used method of abortion [2]. This is not the case in our study. Concerning the post-abortion suites, the post-abortion suites are simple with less than 10 days of hospitalization in 20 cases or 34.48%, complicated with 10 days and more of hospitalization in 30 cases or 51.72% and characterized by maternal death in 8 cases or 13.79%. The maternal mortality rate linked to clandestine abortions is 13.79% in our study milieu.

In the case series of Takongmo *et al*. in Yaoundé, the maternal mortality rate due to clandestine abortions was 15.60% [15], which is higher than ours. According to Lichtenberg *et al*. [17] and according to Elam-Evans *et al*. [18], the maternal mortality rate due to clandestine abortions was 0.0006% in the United States of America, which is much lower than ours. Our high death rate figures can be justified by the lack of medicalization of induced abortion because of our legislation which is so restrictive that many women often resort to life-threatening methods. According to Pélissier, illegal abortion is punishable by heavy prison sentences in many other countries, such as Kenya, Nigeria, Senegal and Uganda, where it is punishable by 14 years in prison for the mother and 7 years for the doctor performing the procedure [19]. In the DRC, the Congolese penal code punishes the mother and her abortionist respectively with 5 to 10 years and 5 to 15 years of penal servitude [20].

The weakness of our study is not to have studied the types of complications of clandestine abortions at the same time while its strength is to be the first to study the epidemiological particularity of clandestine abortions in the hospitals of Kananga in the Province of Central Kasai in DRC.

## Conclusion

It is clear in this study that the frequency of clandestine abortions was about one in twenty-eighth patients with about three in four pregnant women under 30 years old, nulliparity was more concerned, pupils and students as well as the unemployed were more concerned in half of cases, nurses were the abortionists encountered in one in three cases, cervical dilation and curettage constitute the most used abortive method and the rate of maternal mortality linked to clandestine abortion was about one in seven cases. Our results serve as the basis for in-depth studies on clandestine abortions and their complications with a view to improving their management in our environment. The prevention of clandestine abortions must go through the promotion of sex education in school and academic settings, and the increase of awareness campaigns for women and couples on the use of methods of contraception and family planning.

### What is known about this topic

- Clandestine abortion is a voluntary termination of pregnancy under conditions not authorized by law, it has become rare in Western developed countries due to the legalization of induced abortion;
- It is a source of morbidity and especially of maternal mortality in the world;
- The lack of data on the epidemiology for clandestine abortion in hospitals of Kananga, in the DR Congo.

### What this study adds

- The frequency of clandestine abortion is of 3.48% in our milieu;
- Nulliparous pregnant women, under 30 years old, pupils and students as well as unemployed are more concerned, the nurses were the abortionists encountered, cervical dilation and curettage constitute the most used abortive method, and the maternal mortality rate linked to clandestine abortion is 13.79% in our environment;
- The promotion of sex education in schools and universities, and the increase in awareness campaigns for women and couples on the use of methods of contraception and family planning will help reduce clandestine abortions.
